# Scalable, Dual-Band Metasurface Array for Electromagnetic Energy Harvesting and Wireless Power Transfer

**DOI:** 10.3390/mi13101712

**Published:** 2022-10-11

**Authors:** Yiqing Wei, Junping Duan, Huihui Jing, Huimin Yang, Hao Deng, Chengwei Song, Jiayun Wang, Zeng Qu, Binzhen Zhang

**Affiliations:** 1 School of Instrument and Electronics, North University of China, Taiyuan 030051, China; 2 State Key Laboratory of Dynamic Testing Technology, North University of China, Taiyuan 030051, China; 3 School of computer and information engineering, Shanxi Technology and Business College, Taiyuan 030072, China

**Keywords:** ambient energy harvesting (AEH), dual-band rectenna, metasurface array absorber, rectifier, wireless power transfer (WPT)

## Abstract

A dual-band metasurface array is presented in this paper for electromagnetic (EM) energy harvesting in the Wi-Fi band and *Ku* band. The array consists of metasurface unit cells, rectifiers, and load resistors. The metasurface units within each column are interconnected to establish two channels of energy delivery, enabling the transmission and aggregation of incident power. At the terminals of two channels, a single series diode rectifier and a voltage doubler rectifier are integrated into them to rectify the energy in the Wi-Fi band and the *Ku* band, respectively. A 7 × 7 prototype of the metasurface array is fabricated and tested. The measured results in the anechoic chamber show that the RF-to-dc efficiencies of the prototype at 2.4 GHz and 12.6 GHz reach 64% and 55% accordingly, when the available incident power at the surface is 3 dBm and 14 dBm, respectively.

## 1. Introduction

Metamaterials are artificial media with negative permittivity and negative permeability and thus have promising applications in electromagnetic (EM) cloaking, anechoic chamber design, and radar cross-section reduction [1,2,3,4]. With its unique EM properties, the metamaterial absorber achieves the performance of minimizing reflection coefficient and near-zero transmission coefficient, thereby achieving complete absorption of EM energy [5,6,7,8]. However, the energy absorbed in most absorbers is not utilized but rather dissipated in the medium.

Recently, an approach of integrating a rectifier into the metamaterial absorber to fulfill energy conversion has been proposed, which has a promising application in ambient energy harvesting (AEH) and wireless power transfer (WPT). Yet, the very low available power density (μW-mW) in the environment increases the difficulty of energy harvesting [9]. Moreover, the diode rectifier losses have also an influence on efficient energy harvesting. Thus, many approaches have been proposed for reducing energy loss and improving energy collection efficiency. One approach of embedding rectifier diodes directly between the absorber unit cells is proposed to achieve good energy harvesting performance [10,11,12]. Though the energy transfer loss is eliminated by direct impedance matching between the structure unit cell and the diode, it can only be applied to specific metamaterial resonant structures. In addition, another approach for energy transfer by punching holes in the metamaterial unit cells has been proposed [13,14,15]. While this approach achieves efficient energy transfer, additional structural layers need to be added to achieve terminal rectification. Similarly, the method of energy transfer through EM coupling between multiple layers is proposed, which obviously requires multiple layers to implement, and therefore inevitably increases the complexity of fabrication [16]. Furthermore, the energy collector usually also requires additional layers to build the rectification network, regardless of the perforation or EM coupling, which also increases the difficulty of efficient energy collection. Additionally, another approach has been proposed to obtain high energy harvesting efficiency by interconnecting metamaterial unit cells and rectifying them at the terminals [17,18]. The energy captured by the metasurface absorber is transferred to the terminals by interconnecting the unit cells, thus the structure of the energy collector is simplified and the energy loss from the multilayer structure is eliminated. However, the size of the unit cell for energy harvesting is still large and the incident power density is still high in order to obtain high harvesting efficiency.

The aim of this paper is to design a novel dual-band scalable periodic metasurface energy harvester based on small unit size, simple structure, and adaptability to different input power levels. Our design is composed of three parts: a periodic meta surface array, a rectifier, and a load. By interconnecting the structural unit cells of a simple sandwich metamaterial absorber, the radio frequency (RF) energy transmission channels are constructed and the incident power at the terminals is aggregated. Therefore, only a low incident power density is required to achieve efficient energy capture. In addition, the cell is constructed with a common three-layer structure, which does not require additional structural layers to deliver energy. Meanwhile, the designed metasurface unit is also relatively small in size and simple in structure with good scalability. Furthermore, the energy of low and high input power is efficiently converted at different frequencies by integrating a single series rectifier network and a voltage doubler rectifier network at the terminal of the array, respectively. Moreover, the metasurface array is able to maintain high energy harvesting efficiency over a wide range of input powers, whether operating at low or high input power levels. In addition, it can be easily adapted to various power density environments and different sizes of spaces for electromagnetic energy harvesting by reconfiguring the number of the array.

Above all, the structural design and performance analysis of the metasurface array are presented in Section 2. Then, a single series rectifier topology and a voltage multiplier rectifier topology are provided in Section 3 and their performance is simulated separately. Next, Section 4 gives the experimental validation of the metasurface array prototype, and conclusions are presented in Section 5.

## 2. Metasurface Array Analysis and Design

**Figure 1:**
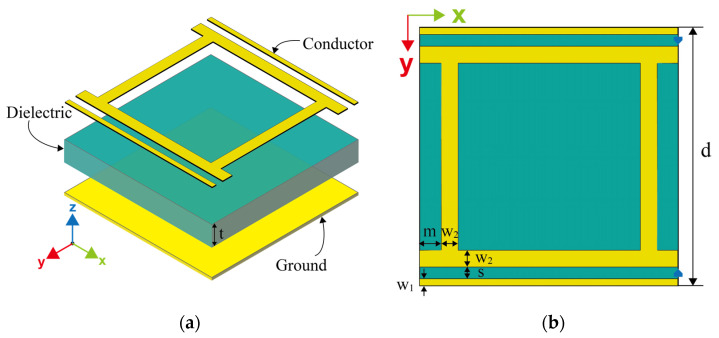
Schematic of the metasurface array unit cell. (**a**) 3-D view. (**b**) Top view.

Metamaterial absorbers are used to achieve complete absorption of incident EM waves at resonant frequencies via resonant structures, loss in the dielectric layer, or by adding resistive loads [19,20]. The proposed metasurface array unit cell is a typical sandwich structure, as shown in Figure 1. The resonant structure of the top layer generates EM resonance at the gap on both sides, with resonant frequencies of 2.4 GHz and 12.6 GHz, respectively. Each side of the dielectric layer is etched with a copper of 0.035 mm thickness, forming the top and bottom layers of the metamaterial absorber. The F4B material with thickness t = 3 mm is used as an intermediate dielectric layer with a dielectric constant of *ε*_r_ = 2.2 and loss tangent of tan δ = 0.0009, respectively. At such a low loss tangent, the energy dissipated on the dielectric layer is almost negligible, which makes it possible for efficient collection of the metasurface energy harvester.

The boundary of both sides of the metasurface unit cell is d = 21 mm. Microstrip lines on both sides of the unit cell, with a width w_1_ of 0.3 mm, are coincident with the boundary, thus the energy transfer channel of the metasurface array is established. The internal patterns of the resonant structure have a width w_2_ of 1 mm and both overlap with the boundary, forming another arm of the energy transfer channel. The distances of both microstrip lines along the *y*-axis from the boundary are m = 0.5 mm, and the distance between the two sides of the gap causing resonance is also s = 0.5 mm. When the incident EM wave resonates at the gap on both sides, the energy is delivered to the load at the terminal along the microstrip lines on both sides of the unit cell. Then, the incident EM energy is completely captured by the metasurface array and concentrated on the load in the event that suitable load resistance is chosen to allow the array to match the spatial impedance.

**Figure 2:**
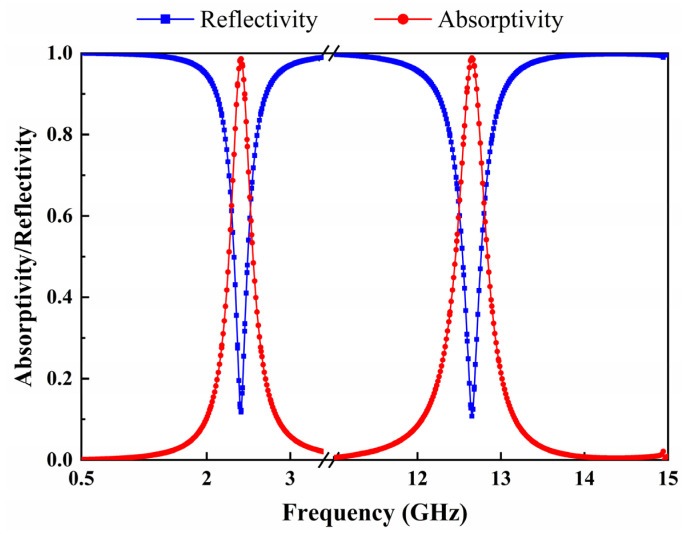
Simulated absorptivity and reflectivity of the metasurface array as a function of frequency.

**Figure 3:**
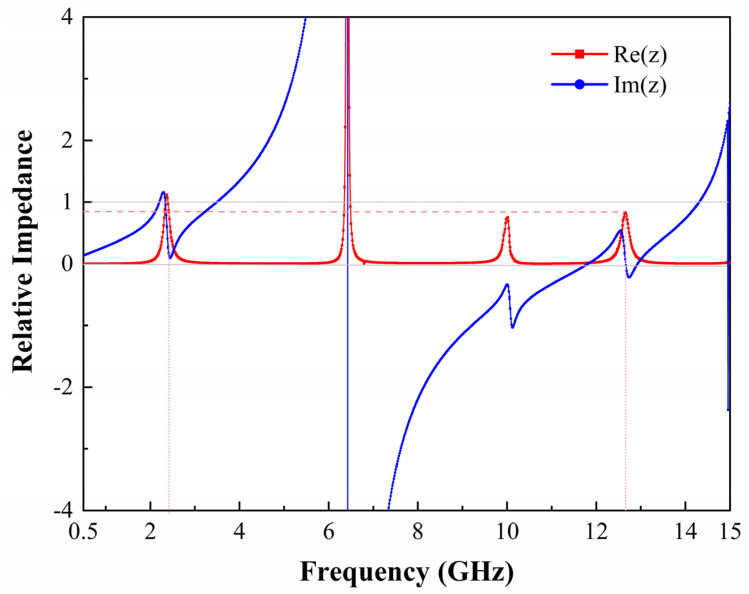
Simulated the real and imaginary parts of the relative input impedance of the metasurface array unit cell.

The proposed metasurface array is simulated by the EM simulation software CST Microwave Studio under periodic boundary conditions with a Floquet port. The unit cell is excited in the *xy* plane by an incident plane wave along the −*z* direction. Figure 2 shows the variation curves of the absorptivity and reflectivity as a function of frequency for the metasurface array when the electric field direction of the incident plane wave is parallel to the *y*-axis. The resistance value of the load located on both sides of the unit cell gap is 900 Ω. It can be observed that the absorptivity of the metasurface array is close to 1 for both resonant frequencies of 2.4 GHz and 12.6 GHz, and the incident EM energy is completely absorbed. It is also demonstrated by the relative input impedance curves of the metasurface array at resonant frequencies shown in Figure 3. It can be noticed that the real part of the relative input impedance of the array is close to 1 and the imaginary part is close to 0 at both 2.4 GHz and 12.6 GHz. It shows that the metasurface array unit cell has good impedance matching at both resonant frequencies when terminated by a 900-Ω load and therefore has good absorption performance.

**Figure 4:**
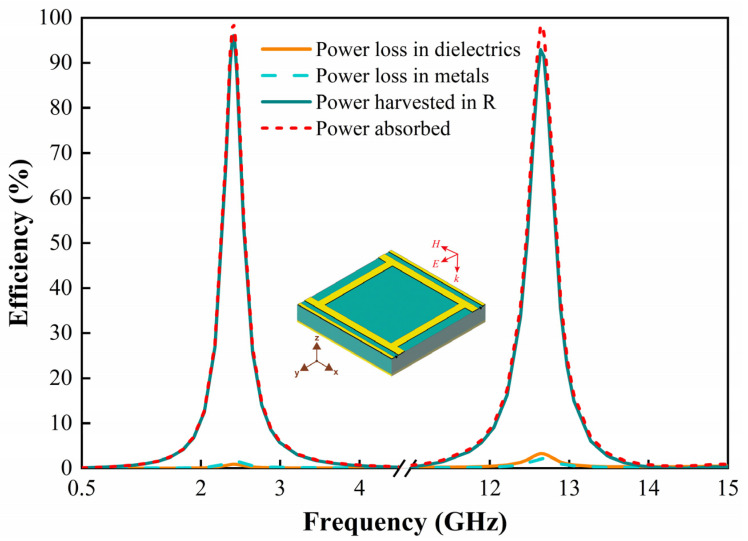
The power absorption, loss, and harvesting efficiency of the metasurface array under incident plane wave irradiation in the −*z*-direction.

Next, the dissipated power distribution of each element of the metasurface array is analyzed. Figure 4 shows the overall absorption efficiency of the unit cell and the harvesting efficiency on the metal, dielectric layer, and resistive load when the incident electric field travels along the *y*-axis. The radiation-to-AC conversion efficiency of the metasurface array is determined by

(1) $$\eta_{\mathrm{Rad}\text{-}\mathrm{ac}}=\frac{P_{\mathrm{ac}}}{P_{\mathrm{rad}}}$$

where *P*_ac_ is the absorbed power across the resistive load and *P*_rad_ is the available incident power over the physical area of the unit cell. As can be seen in Figure 4, the energy absorbed by the metasurface array is mainly concentrated in the load resistance achieving 96% and 92.9% at the resonant frequencies of 2.4 GHz and 12.6 GHz, respectively. While the energy dissipated in the metal and the dielectric is almost negligible, which makes it possible to collect the incident energy efficiently.

**Figure 5:**
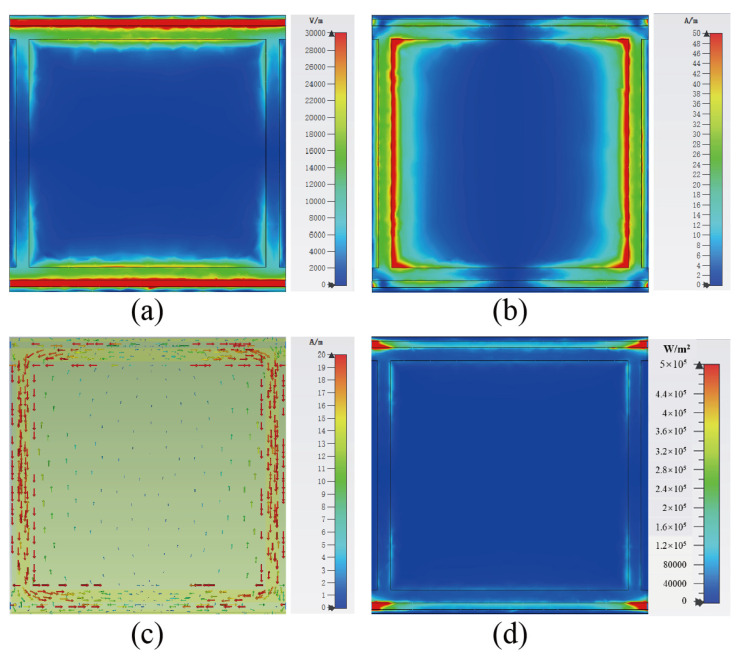
Simulations of (**a**) electric field magnitude, (**b**) magnetic field magnitude, (**c**) surface current distribution, and (**d**) power flow diagram distribution of the metasurface array unit cell at 2.4 GHz with incident plane wave irradiation along the −*z*-direction.

**Figure 6:**
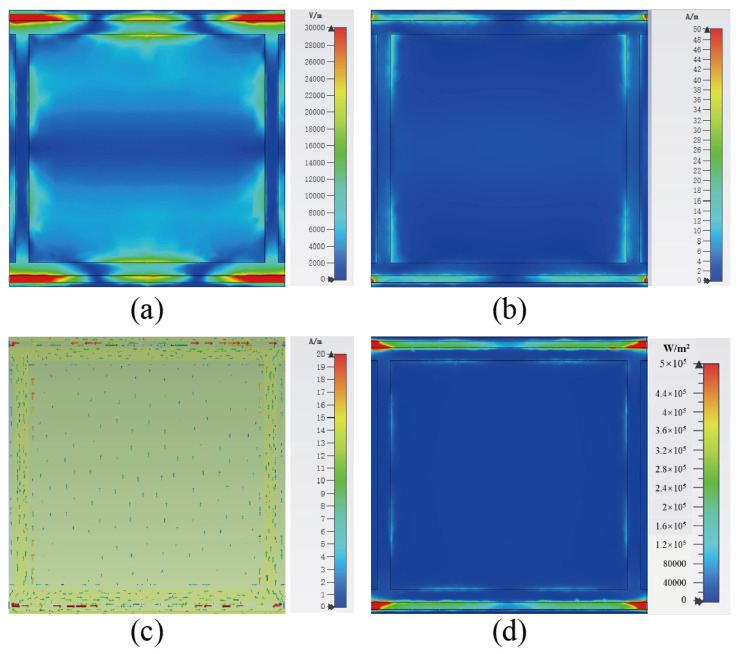
Simulations of (**a**) electric field magnitude, (**b**) magnetic field magnitude, (**c**) surface current distribution, and (**d**) power flow diagram distribution of the metasurface array unit cell at 12.6 GHz with incident plane wave irradiation along the −*z*-direction.

Finally, we analyzed the electric field, magnetic field, surface current, and power flow distribution of the metasurface array at two resonant frequencies, as shown in Figure 5 and Figure 6. Figure 5 presents the distribution of the electrical properties of the metasurface unit cell at 2.4 GHz. It can be seen that the EM resonance occurs mainly at the gap on both sides of the unit cell at 2.4 GHz, as shown by the electric field distribution in Figure 5a. The strong electric field distribution on both sides of the gap indicates that there is a larger charge accumulation at the edges of the microstrip lines. The microstrip lines on both sides of the gap can be described by the inductance, while the gap can be expressed as the capacitance. Therefore, LC resonant circuit theory can be used to illustrate the resonant absorption of the metasurface array [21]. The magnetic field of the unit cell is mainly distributed on the two branches along the *y*-axis, as shown in Figure 5b. Accordingly, the surface current of the unit cell flows along the two branches of the *y*-axis, as shown in Figure 5c. Finally, the power flow distribution at 2.4 GHz is shown in Figure 5d. It can be observed that the energy captured by the metasurface array at 2.4 GHz is mainly concentrated in the load.

Figure 6 shows the distribution of electrical properties of the metasurface array at 12.6 GHz. It can be seen from the electric and magnetic field distributions in Figure 6a,b that the EM coupling of the metasurface array is mainly contributed by the gap on both sides of the unit cell in the *x*-axis direction and the two branches in the *y*-axis direction. The resonance at higher resonant frequencies can be explained by the fundamental dipole mode [22,23,24]. Since the dipole resonance has a large local charge distribution on both sides of the junction, the electric field can excite an enhanced local electromagnetic field inside the junction. The surface currents flow along the branches in the *x*-axis and *y*-axis directions, respectively, and cross the load resistance, as shown in Figure 6c. Additionally, the power flow distribution is concentrated at both ends of the load resistor in Figure 6d under 12.6 GHz, the same as in Figure 5d, which are all consistent with the findings in Figure 4 where the energy is mainly captured by the resistive load.

## 3. Dual-Band Rectifier Design

The rectifier, as a component of the energy conversion of the metasurface energy harvester, is usually composed of a rectifier diode and an impedance matching circuit to achieve efficient energy transfer and conversion. According to the connection of the rectifier diodes, rectifiers can be sorted into the single series diode rectifier [25,26], the single parallel diode rectifier [27], the diode bridge rectifier [28], and the voltage doubler rectifier [29], etc. In conventional rectifier designs, the impedance matching networks used are usually different for the different types of diodes, which is caused by the nonlinearity of the diodes and the various packaging technologies. Yet, the proposed metasurface array unit cell is characterized by high input impedance at the terminal, 900 Ω to achieve the perfect matching, and thus can achieve good matching with different types of diode rectifiers. Two energy transfer channels are then established along the *x*-axis of the metasurface array shown in Figure 1 so that energy harvesting with different frequencies and different input powers can be achieved by connecting different types of diode rectifiers to them.

**Figure 7:**
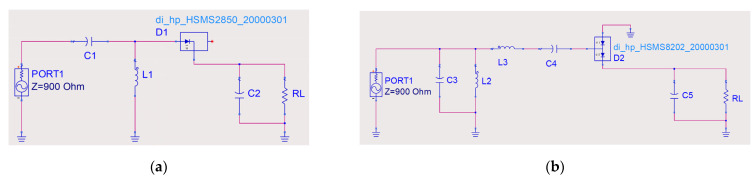
Schematic of the proposed voltage doubler rectifier in ADS. Schematic diagram of (**a**) the single series diode rectifier and (**b**) the voltage multiplier rectifier proposed in the ADS. Here, C1 = 4 pF, C2 = C5 = 1000 pF, C3 = C4 = 0.2 pF, L1 = 22 nH, L2 = 2.2 nH, L3 = 1.5 nH, (**a**) RL = 1000 Ω, and (**b**) RL = 600 Ω.

Figure 7a,b show the topologies of the single series diode rectifier and the voltage doubler rectifier in the Advanced Design System (ADS), respectively. The rectifier shown in Figure 7a is designed to rectify at 2.4 GHz because of its high rectification efficiency at low input power levels. The Schottky diode Avago HSMS-2850 was chosen due to its lower turn-on voltage characteristics, enabling a high rectification efficiency at low input power levels [25,26]. C1 and L1 were used to form an L-shaped impedance matching network for efficient energy transfer. The voltage drop across the load resistor RL can be a monitor of the rectifier’s performance. The capacitor C2, connected in parallel, was used to smooth the output waveform and store DC power. Figure 7b shows a voltage doubler rectifier for rectification of incident signals at high input power levels under 12.6 GHz. The Schottky diode Avago HSMS-8202, suitable for operation at high input power, was selected as the main component of the rectifier. As shown in Figure 7b, the topology of L2 and C3 in parallel and L3 and C4 in series constitutes an LC bandpass impedance matching circuit. In addition, C4 also acts as a DC blocker apart from being a part of the impedance matching network. The capacitor C5 is used to smooth the output ripple and ensure that the DC passes to the load.

**Figure 8:**
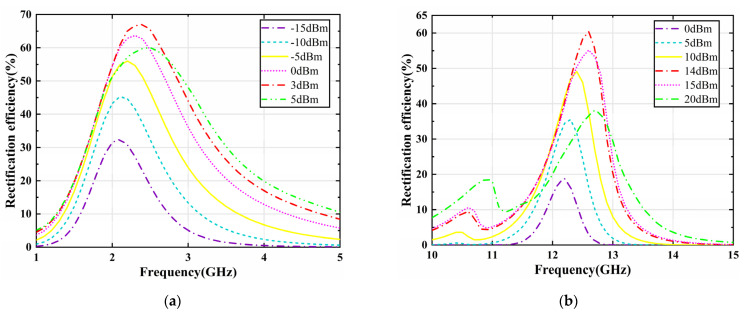
Simulated rectification efficiency curves versus frequency for (**a**) the single series diode rectifier and (**b**) the voltage doubler rectifier at different input power levels.

**Figure 9:**
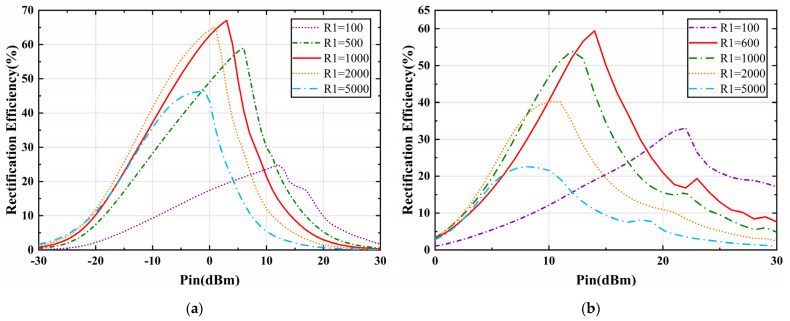
Simulated rectification efficiency of rectifiers versus input power at various loads. (**a**) 2.4 GHz. (**b**) 12.6 GHz.

**Figure 10:**
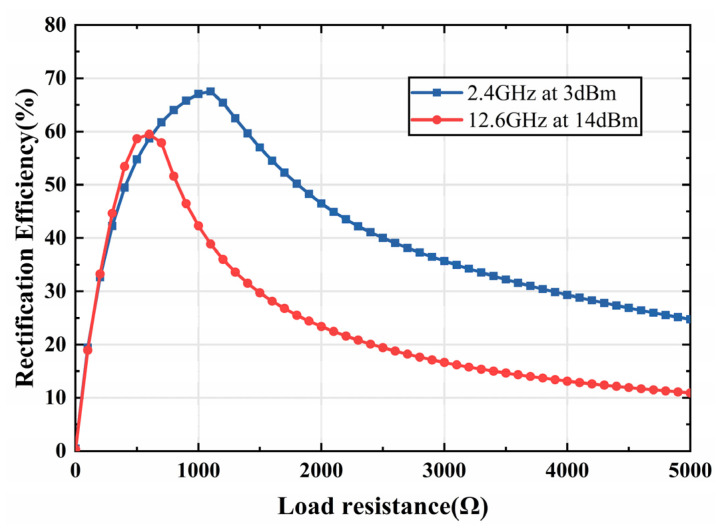
Simulated rectification efficiency curves versus load resistance for the single series diode rectifier and the voltage doubler rectifier.

The proposed metasurface array is interconnected by energy transfer channels along the *x*-axis and connected to the rectifier at the terminals, whereby the energy from the end of a row of unit cells is aggregated and captured, resulting in an increase in the incident power. As for the nonlinear variation of the input impedance of the diode with the input power and frequency, we have simulated and optimized the rectifier circuits in Figure 7a,b, respectively, with the harmonic balance (HB) solver in ADS. The overall performance of the rectifier circuits for the metasurface array at two frequencies is shown in Figure 8, Figure 9 and Figure 10. The variation curves of rectification efficiency with frequency for different input power levels are shown in Figure 8a,b, respectively. Rectification efficiency can be given as

(2) $$\eta_{\mathrm{ac}\text{-}\mathrm{dc}}=\frac{P_{\mathrm{dc}}}{P_{\mathrm{ac}}}$$

where *P*_dc_ is the output power at the load resistor of the rectifier output terminal and *P*_ac_ is the incoming power of the rectifier. It can be observed that the rectification efficiency of the single series diode rectifier at 2.4 GHz reaches a maximum of 67.1% when the incident power is 3 dBm, as shown in Figure 8a. While the rectification efficiency of the voltage doubler rectifier at 12.6 GHz reaches the maximum of 60.4% at an incident power of 14 dBm.

Furthermore, the variation of rectification efficiency versus input power and load resistance is analyzed as shown in Figure 9a,b. It can be noticed that the rectification efficiency grows with the increase of input power at both 2.4 GHz and 12.6 GHz and reaches the maximum at 3 dBm and 14 dBm, respectively. In this case, the efficiency reaches the maximum at 2.4 GHz and 12.6 GHz for load resistors RL with values of 1000 Ω and 600 Ω, respectively. The variation curve shown in Figure 9 well illustrates the nonlinearity of the rectifier diode against the input power and load resistance, which is directly related to whether the rectifier can be well matched. Finally, the rectification efficiency versus load impedance is analyzed for the given input power, as shown in Figure 10. It can be seen that the rectification efficiency of the two rectifiers gradually reaches its maximum and then gradually drops as the load resistance value increases. Such results are consistent with the maximum efficiency of Figure 8 and Figure 9, which also verify the validity of the impedance matching network and rectifier circuit design.

## 4. Measurement and Discussion

**Figure 11:**
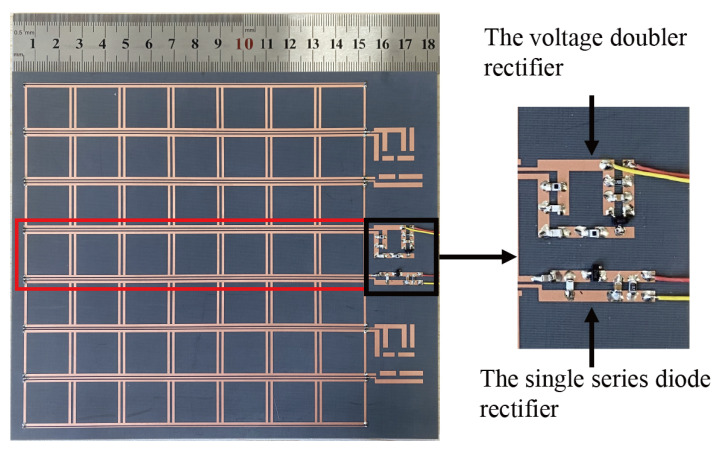
Fabricated metasurface array with integrated rectifier.

A 7 × 7 metasurface resonant structure is plated on a 3-mm F4B dielectric substrate with 35-μm copper, as shown in Figure 11. The footprint area of the prototype dielectric layer is 182 mm × 157 mm, while both the top and bottom layers have a copper cladding area of 147 mm × 147 mm. Seven unit cells in each column of the fabricated prototype along the *x*-axis are interconnected to form an energy flow channel. The middle column of the array was tested with a single series diode rectifier and a voltage doubler rectifier, which were integrated with the dual-channel terminals, respectively. The reason for choosing the middle column for testing is to minimize the effect of non-uniform coupling. The remaining six columns of the array are terminated with 900-Ω resistors.

**Figure 12:**
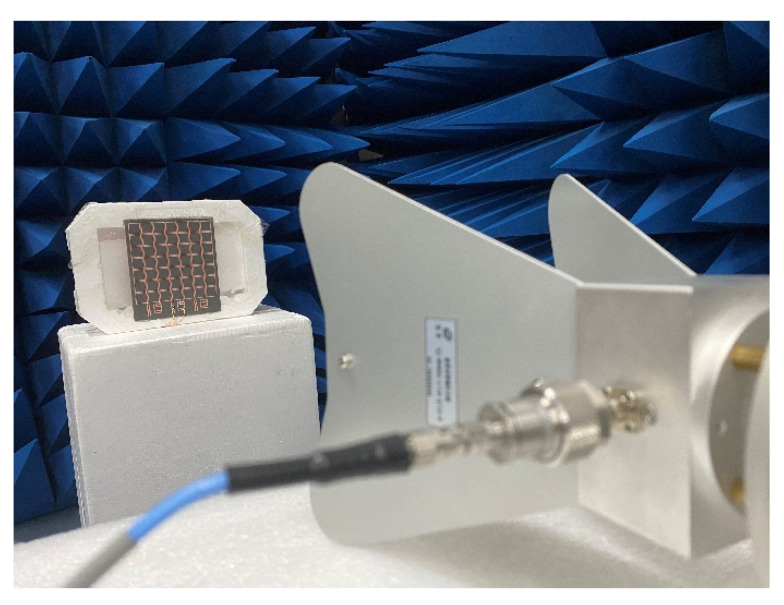
Photograph of metasurface array being measured under broadband horn antenna illumination in the anechoic chamber.

As shown in Figure 12, the measurement of the metasurface array prototype was performed in an anechoic chamber. First, the RF signal produced by the signal generator was transmitted to the standard horn antenna covering the measurement band. Second, the desired incident plane wave was illuminated by the horn antenna onto the prototype situated at the far-field position. Finally, the dc output voltage across the load resistor on the prototype terminal was recorded by a digital multimeter. The overall efficiency of the metasurface array energy harvesting can be exhibited as follows:

(3) $$\eta_{mea.}=\frac{P_{\mathrm{dc}}}{P_{\mathrm{in}}}$$

where *P*_dc_ is the output power across the rectifier load resistor, and *P*_in_ is the total available incident power absorbed in the surface region of the array, respectively, shown as follows:

(4) $$P_{\mathrm{dc}}=\frac{V_{\mathrm{out}}^{2}}{R_{\mathrm{load}}}$$

(5) $$P_{\mathrm{in}}=\frac{G_{t}\cdot P}{4\pi R^{2}}\cdot A_{s}$$

where *V*_out_ represents the dc voltage landing on the rectifier load *R*_load_. *G_t_* denotes the gain of the horn antenna and *P* represents the power excited by the signal source. *R* is denoted as the distance from the horn antenna to the prototype. *A_s_* represents the effective receiving aperture of the metasurface array, which can be given by the physical footprint of the middle column under the test.

**Figure 13:**
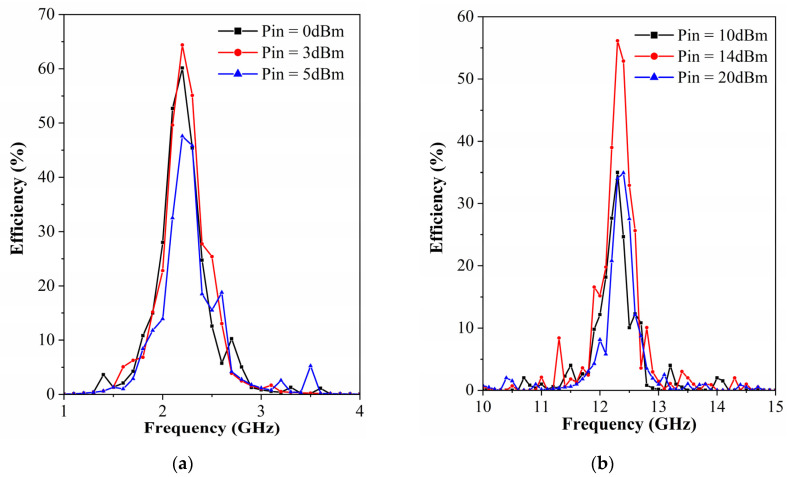
Measured RF-to-dc efficiency of the proposed metasurface array as a function of frequency with (**a**) the single series diode rectifier and (**b**) the voltage doubler rectifier.

As the fabricated prototype is irradiated by a normal incident plane wave along the −*z* direction, the RF-to-dc efficiency versus frequency at different available power levels is shown in Figure 13. As can be seen from Figure 13a, the metasurface array with an integrated single series diode rectifier achieves a maximum conversion efficiency of 64% at 2.2 GHz when the incident available power is 3 dBm. When the incident available power is 14 dBm, the conversion efficiency of the metasurface array with an integrated voltage doubler rectifier reaches a maximum of 55% at 12.3 GHz.

It can also be observed in Figure 13 that the manufactured prototype undergoes a frequency shift and a reduction in conversion efficiency. It is firstly due to the conversion efficiency of the prototype being highly dependent on the rectification performance of the rectifier circuit compared to the absorption efficiency of the metasurface array. Secondly, the non-uniform coupling among the finite number of metasurface array units is also a very important factor. Although the middle column of the prototype was tested, it inevitably caused a bias in the test results compared to the simulation of the infinite period metasurface. Finally, manufacturing deviations, whether caused by the 7 × 7 metasurface array panel or the layout of the rectification network and the soldering of the circuits, will cause frequency shifts and a reduction in conversion efficiency.

**Figure 14:**
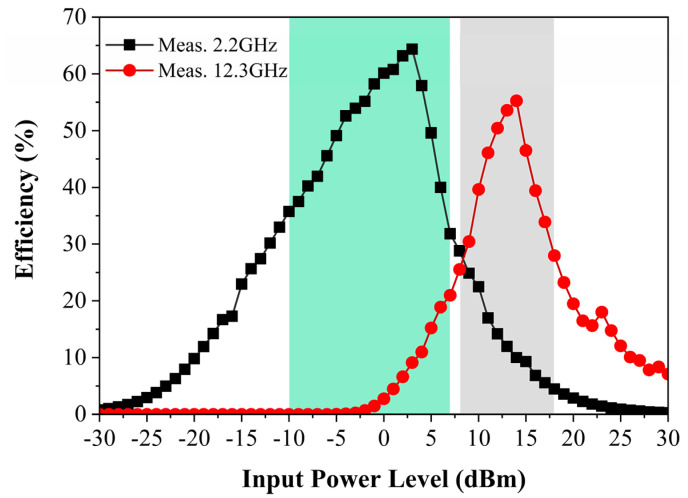
Measured RF-to-dc efficiency of the proposed metasurface array versus input power levels at 2.2 GHz and 12.3 GHz.

Next, the conversion efficiency of the metasurface array at 2.2 GHz and 12.3 GHz was measured separately as a function of the available input power at the surface, as shown in Figure 14. It can be observed that the conversion efficiency of the prototype at 2.2 GHz reaches 64% at 3 dBm when the load of the single series diode rectifier is 1000 Ω. Similarly, the conversion efficiency of the prototype reaches 55% at 14 dBm under 12.6 GHz when the load of the voltage doubler rectifier is 600 Ω. In addition, the metasurface array can still maintain high harvesting efficiency at a wide available power of −10–7 dBm at 2.2 GHz and a wide available power of 8–18 dBm at 12.3 GHz, respectively.

Based on simulations and measurements, the proposed metasurface array is compared with previous energy harvesting works reported in the literature, as shown in Table 1. It can be seen that a new design based on a small unit size, a simple structure, and adaptability to different input powers for a dual-band metasurface is proposed in the article. In comparison with other related work, the metasurface array avoids the integration of rectifier diodes in each cell by a built-in channel approach, which simplifies the number of structural layers and reduces the difficulty of fabrication. Moreover, the feature of interconnecting adjacent units to aggregate energy enables the metasurface array to achieve high harvesting efficiency at low input power. Furthermore, two channels of each column are integrated by a single series diode rectifier and a voltage doubler rectifier, respectively, to achieve high conversion efficiency at different frequencies with different input powers. In addition, the relatively small size of the designed unit makes it easy to expand, which can be easily adapted to different space sizes for energy harvesting. It is also to be noted that the metasurface array can maintain a high energy harvesting efficiency over a wide range of input power, whether it operates at low or high input power levels. Therefore, the proposed metasurface array has better adaptability in AEH and WPT compared to other works.

## 5. Conclusions

A dual-band metasurface array operating in the Wi-Fi band and Ku band is designed and optimized. Two energy channels are constructed by interconnecting the units, enabling the transmission and aggregation of energy, which in turn increases the power density of the channel terminals. A single series diode rectifier is integrated at the terminal of one channel, thus enabling the efficient collection of low input power at 2.4 GHz. At the terminal of another channel, a voltage doubler rectifier is integrated to achieve high input power collection at 12.6 GHz. Then, a 7 × 7 metasurface array is fabricated. The test results show that the RF-to-dc efficiency of the prototype reaches 64% and 55% at 2.2 GHz and 12.3 GHz, respectively, with the available incident power at the surface of the array correspondingly at 3 dBm and 14 dBm. Moreover, the proposed metasurface array is capable of maintaining a high collection efficiency over a wide range of input power levels at both dual-band resonant frequencies. Additionally, the proposed metasurface array possesses the characteristics of simple design and a scalable number of units, thus allowing efficient energy harvesting in different environments.

## Figures and Tables

**Table 1:** Comparison of the proposed metasurface array with related works.

Reference	Frequency (GHz)	Diodes to Each Cell	Dimension of Unit Cell (mm)	Prototype Size (mm)	RF-to-dc Efficiency (%)
[16]	Single-band 5.8	No	31.7 × 31.7 (5 layers)	190.2 × 190.2 (array: 6 × 6)	55%@35 μW/cm^2^
[17]	Single-band 2.45	Yes	35 × 57.5 × 6.5 (3 layers)	228.6 × 304.8 (array: 5 × 6)	61%@15 dBm
[26]	Dual-band 0.94, 1.84	NA	NA	240 × 240	42%@0.14 μW/cm^2^
[28]	Single-band 2.45	No	74 × 67 × 4.769 (5 layers)	229 × 305 (array: 3 × 4)	61%@313 μW/cm^2^
[30]	Dual-band 2.4, 5.8	Yes	16 × 15.5 × 1.27 (3 layers)	67 × 64 (array: 4 × 4)	58%, 51%@0 dBm
[31]	Single-band 2.84	Yes	50 × 50 × 3.175 (3 layers)	200 × 200 (array: 4 × 4)	60%@187.5 μW/cm^2^
[32]	Single-band 2.45	Yes	20 × 20 × 4.2 (6 layers)	120 × 120 (array: 6 × 6)	66.9%@5000 μW/cm^2^
This work	Dual-band 2.4, 12.6	No	21 × 21 × 3.07 (3 layers)	182 × 157 (array: 7 × 7)	64%@3 dBm 2.2 GHz, 55%@14 dBm 12.3 GHz
